# Pacific Island Countries and Climate Change: Examining Associated Human Health Vulnerabilities

**DOI:** 10.1289/ehp.124-A208

**Published:** 2016-11-01

**Authors:** Nancy Averett

**Affiliations:** Nancy Averett writes about science and the environment from Cincinnati, OH. Her work has been published in *Pacific Standard*, *Audubon*, *Discover*, *E/The Environmental Magazine*, and a variety of other publications.

Climate change presents a significant and growing threat to human health, with diverse impacts projected for different regions.^1^ Investigators now report that Pacific island countries including Fiji, Tonga, and the Marshall Islands are among the nations most vulnerable to climate-related health problems due to their particular geographic, demographic, and socioeconomic characteristics.^2^ Their new paper is a synthesis of the key technical findings and policy implications of the 2015 World Health Organization report *Human Health and Climate Change in Pacific Island Countries*, written by the same group.^3^

**Figure:**
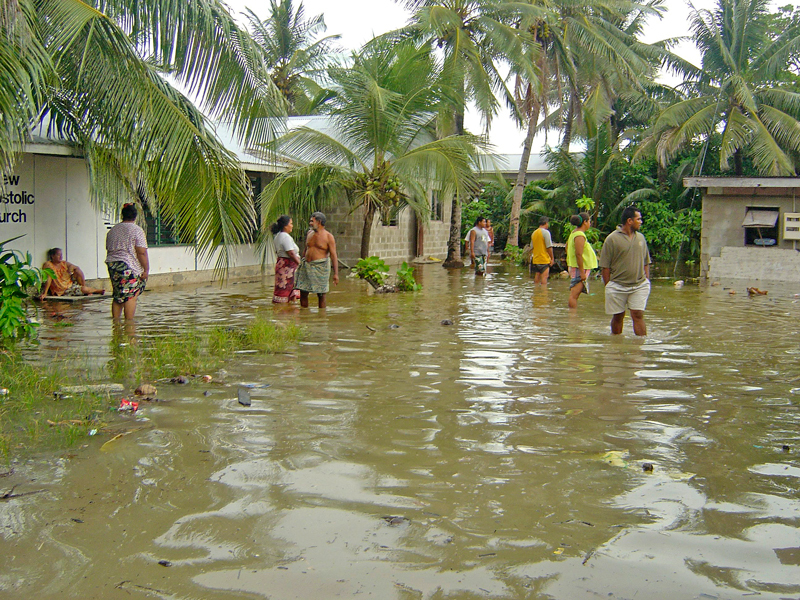
An unusually high “king tide,” one of the highest of the past 25 years, swamped Tuvalu in February 2006. A combination of sea-level rise and larger storm-driven waves is expected to flood such low-lying Pacific island countries with increasing frequency.^7^ © Philippe Petit/Paris Match via Getty Images

First author Lachlan McIver, an associate professor in the College of Public Health, Medical, and Veterinary Sciences at Australia’s James Cook University, says that when teams of climate change and health consultants began their assessment in 2011, not many regions or countries had undertaken vulnerability and adaptation assessments or been able to derive results and act upon them, “so we were really on a bit of a crest of the wave in that sense.” He says the teams found that not all “best practices” described in the literature for assessing climate change health vulnerabilities actually worked in practice in the Pacific island countries due, in part, to a lack of data in some countries. Thus, he says, the consultants found they had to be flexible and use both quantitative and qualitative methods in their research and analysis.

The authors examined 13 Pacific island countries in terms of 3 categories of climate-related health concerns that they termed “direct,” “indirect,” and “diffuse.” Direct effects included physical and psychological trauma related to an extreme weather event such as a hurricane or a heat wave. Indirect effects included increased burdens of disease resulting from climate-related disruption—for instance, a rise in vector-borne diseases if ecological disruption were to create conditions favorable to the spread of pathogen-carrying pests. Finally, diffuse effects included increased mental health problems, injuries, and violent deaths that could result as societal dysfunction unfolds; this unfolding would be due to such phenomena as loss of livelihood or a lack of basic resources including water, food, and housing.^2^

The teams worked with stakeholders in each country to develop lists of their highest-priority climate-sensitive health risks, then decide which ones to address in their adaptation plans. Some countries chose to include all relevant risks; others picked just those deemed to be the greatest threat. Because of that variation, the report contains this caveat: “The climate-sensitive health risks presented … should be considered a synthesis of each country’s priorities rather than a true cross-country comparison of risks.”^2^

Most countries placed water security, food security, vector-borne diseases, and direct health impacts of extreme weather events among their top priorities. Pacific island populations also face a unique climate-related health risk in terms of their extremely high levels of noncommunicable diseases, including obesity, diabetes, and hypertension. Noncommunicable diseases are already leading causes of death in these populations,^4^ partly because of a high dependence on energy-dense, high-calorie imported foods rather than locally grown products.^5^ In an example of a diffuse effect, climate change could exacerbate these trends because higher temperatures, altered rainfall patterns, and sea level rise will make it even more difficult to grow local food; increased reliance on imported foods could, in turn, lead to food insecurity.^2^

Kathryn Bowen, a senior research fellow at the Australian National University, says the work was an important first step. “Until we do these types of vulnerability assessments, we don’t know what to respond to,” says Bowen, who was not involved in the study but has done similar work in other parts of the world. “We don’t know what problems and policies are in place and what additional work needs to be done.”

Colin Butler, a professor of public health at the University of Canberra who was not involved in the study, is concerned with the teams’ method, pointing out that it could result in vital health-related issues not being addressed. “Only Kiribati identifies ‘population pressure’ as a ‘high-priority climate-sensitive health risk,’” he explains, but “demographic pressures are problematic in far more Pacific island countries than Kiribati.” Butler has co-authored papers about how high birth rates in developing countries heighten vulnerability to climate change.^6^

Still, such issues may one day make the priority list of more island countries—the authors point out that their assessment is only the first step in a long journey, and they expect the countries’ adaptation plans to go through frequent revisions in the future. McIver says, “Each country would likely benefit from further support both in doing more sophisticated analysis of climate-sensitive health risks and climate variability and change, and in considering ways those adaption plans can be incorporated into the health and other sector policies.”

For coauthor Kristie Ebi, a professor of environmental and occupational health science at the University of Washington, the concern is whether there will be enough outside funding to help these nations implement their plans. “These islands are suffering the consequences of climate change, and they’re not responsible for it,” she says. “Their total greenhouse gas emissions are tiny … so to ask them to take on [the health burdens associated with climate change] without additional funding really isn’t fair.”

## References

[1] McMichael AJ Lindgren E Climate change: present and future risks to health, and necessary responses. J Intern Med 270 5 401 413 2011, doi:10.1111/j.1365-2796.2011.02415.x 21682780

[2] McIver L Health impacts of climate change in Pacific island countries: a regional assessment of vulnerabilities and adaptation priorities. Environ Health Perspect 124 11 1707 1714 2016, doi:10.1289/ehp.1509756 PMC5089897 26645102

[3] McIver L (2015). Human Health and Climate Change in Pacific Island Countries.. http://iris.wpro.who.int/bitstream/handle/10665.1/12399/9789290617303_eng.pdf?ua=1.

[4] Mannava P Health systems and noncommunicable diseases in the Asia-Pacific region: a review of the published literature. Asia Pac J Public Health 27 NP1 19 2015, doi:10.1177/1010539513500336 24097936

[5] Parry J Pacific islanders pay heavy price for abandoning traditional diet. Bull World Health Org 88 7 484 485 2010, doi:10.2471/BLT.10.010710 20616964 PMC2897991

[6] Bryant L Climate change and family planning: least-developed countries define the agenda. Bull World Health Org 87 11 852 857 2009, doi:10.2471/BLT.08.062562 20072771 PMC2770281

[7] Storlazzi CD Many atolls may be uninhabitable within decades due to climate change. Sci Rep 5 14546 2015, doi:10.1038/srep14546 26403195 PMC4585922

